# Role of limonin in anticancer effects of *Evodia rutaecarpa* on ovarian cancer cells

**DOI:** 10.1186/s12906-020-02890-y

**Published:** 2020-03-20

**Authors:** Jae Ryul Bae, Wook Ha Park, Dong Hoon Suh, Jae Hong No, Yong Beom Kim, Kidong Kim

**Affiliations:** Department of Obstetrics and Gynecology, Seoul National University Bundang Hospital, Seongnam, Republic of Korea

**Keywords:** *Evodia rutaecarpa*, Limonin, p53, Apoptosis, Cisplatin resistance

## Abstract

**Background:**

Ovarian cancer therapy generally involves systemic chemotherapy with anticancer drugs; however, chemotherapy with a platinum-based drug has often been shown to cause adverse reactions and drug resistance in ovarian cancer patients. *Evodia rutaecarpa* (ER) reportedly shows anticancer activity against various types of cancer cells. However, the effects of ER have not yet been fully uncovered in ovarian cancer.

**Methods:**

In the present study, we investigated the anticancer effects of an ER extract and its components against the ovarian cancer cell lines SKOV-33, A2780, RMUG-S and a cisplatin-resistant SKOV-3 cell line (Cis^R^ SKOV-3). Cell viability and colony formation assays along with subcellular fractionation analysis, immunoblotting, and immunofluorescence staining were performed.

**Results:**

ER treatment led to a significant reduction in the viability of SKOV-3 cells. Moreover, limonin, a compound found in ER, reduced the viability of both serous-type (SKOV-3 and A2780) and mucinous-type (RMUG-S) ovarian cancer cells by inducing apoptosis via activation of the p53 signaling pathway. Furthermore, limonin reversed the drug resistance through activation of apoptosis in Cis^R^ SKOV-3.

**Conclusion:**

Taken together, our findings suggest that limonin contributes to the anti-ovarian cancer effects of ER by inducing apoptosis via activation of the p53 signaling pathway.

## Background

Globally, ovarian cancer is the 7th most common cancer and the 8th leading cause of cancer mortality in women [1]. The initial treatment for ovarian cancer involves surgery, followed by chemotherapy. Despite the high response rate to first-line chemotherapeutic drugs, recurrence of ovarian cancer occurs in many cases. Moreover, in patients with ovarian cancer, chemotherapy with existing anticancer drugs, such as cisplatin, leads to adverse reactions and the development of chemoresistance. Adverse reactions, together with chemoresistance, contribute to the failure of anticancer therapy. To address these problems, development of drugs using natural products has been on the rise, as these compounds are considered novel candidate therapeutic agents to treat several types of cancer, with multiple therapeutic effects and few side effects [2, 3].

*Evodia rutaecarpa* (ER), an oriental medicine, has traditionally been used for the treatment of headaches, gastrointestinal diseases, amenorrhea, and postpartum hemorrhage [4–6]. An analytical study on the chemical composition of ER has reported that the plant contains alkaloids, carboxylic acids, essential oils, flavonoids, and limonoids [7]. Several studies have reported that ER and its derivatives exhibit multiple biological activities, including anti-inflammatory, anti-obesity, antihypertensive, and anti-allergic effects [8–10]. Recently, two studies have reported that the activation of caspases and AMP-activated protein kinase by an ethanol extract of ER led to apoptosis of cervical cancer cells and benign prostatic hyperplasia epithelial cells, respectively [11, 12]. The finding that the ER extract inhibits proliferation in various cell lines indicates that the plant or its components may have anticancer activity.

Limonin, one of the compounds found in ER [13, 14], is the major limonoid and a bitter compound, mainly found in seeds. Several studies have indicated that limonin shows biological activities, including antioxidant, anti-inflammatory, and antiviral effects [15–17]. Validation studies have demonstrated the anticancer effects of limonin in various cancer cell lines [18–23]. Mechanistic investigations have shown that limonin inhibits cell growth by inducing apoptosis. For example, both hepatoma HepG2 and colon cancer SW480 cells were shown to exhibit increased levels of proapoptotic proteins, including Bax and caspase-3, with limonin treatment [18, 19]. Moreover, limonin exhibited cytotoxicity toward a human breast cancer cell line, MCF-7, via activation of caspase-7, without disrupting the activity of aromatase [20]. Thus, numerous studies have shown that limonin exerts common anticancer effects against various cancer cell lines, suggesting that it has a therapeutic potential for treating various cancers. However, there is limited evidence regarding the anti-ovarian cancer effects of ER and limonin.

Hence, in this study, we explored the pharmacological potential of ER against ovarian cancer and the role of limonin in the anticancer effects of ER.

## Methods

### Cell culture and reagents

SKOV-3 and A2780, human ovarian cancer cell lines of serous histology, and RMUG-S, a human ovarian cancer cell line of mucinous histology, were obtained from the American Type Culture Collection (Rockville, MD, USA) and the Japanese Collection of Research Bioresources Cell Bank (Osaka, Japan), respectively. The serous-type cell lines, SKOV-3 and A2780, were cultured in Roswell Park Memorial Institute 1640 medium (Welgene, Kyungsan, Republic of Korea) containing 10% fetal bovine serum and 1% penicillin–streptomycin (Invitrogen, Carlsbad, CA, USA), and the mucinous-type cell line, RMUG-S, was cultured in DMEM/F12 (Sigma–Aldrich, St. Louis, MO, USA) containing 10% fetal bovine serum and 1% penicillin–streptomycin in a humidified incubator at 37 °C with 5% CO_2_.

A water extract of ER was obtained from the National Development Institute of Korean Medicine (Kyungsan, Republic of Korea), and synephrine and limonin were purchased from ChemFaces (Wuhan, China). DMSO (Sigma–Aldrich) was used to dissolve the ER extract, synephrine, and limonin.

### Generation of a cisplatin-resistant (Cis^R^) cell line

To generate Cis^R^ cells, we followed previously reported methods [24], with slight modifications. Briefly, the IC_50_ value of cisplatin (Sigma–Aldrich) against the SKOV-3 cell line was determined by incubating cells with cisplatin (0.01–100 mM) for 72 h and plotting a concentration-response curve. The determined IC_50_ value of cisplatin was used in subsequent experiments. After 72 h, the medium was changed to a fresh medium, without cisplatin, to recover the cells, and then the Cis^R^ subline was continuously maintained for 6 months, according to the developmental protocol. After the developmental period, a new IC_50_ value was determined in a concentration-response experiment with cisplatin, and Cis^R^ cells were maintained with a concentration of cisplatin equal to the new IC_50_ for a further 6 months.

### Cell viability assay

The cell viability assay was performed using the PrestoBlue™ reagent (Invitrogen), according to the manufacturer’s manual. Serous or mucinous ovarian cancer cells were seeded into a 96-well plate (1000 cells per well in growth media) and cultured to approximately 60–70% confluence. The ER extract, synephrine, and limonin, with and without pifithrin-α (PFT-α; Selleckchem, Houston, TX, USA), were added to the seeded cells, and the cells were incubated for 24, 48, and 72 h. Subsequently, a diluted PrestoBlue reagent was added to each well, and the plate was incubated for 2 h in a humidified incubator at 37 °C with 5% CO_2_. Afterward, absorbance was measured at 540 nm using a microplate reader.

### Colorimetric caspase-3 assay

The caspase-3 activity assay was performed using a caspase-3/CPP32 colorimetric assay kit (BioVision, Milpitas, CA, USA) according to the manufacturer’s recommended protocols. Briefly, ER-, synephrine-, and limonin-treated cells were lysed in a cell lysis buffer. Subsequently, the protein concentrations were determined in the lysates using a BCA assay (Thermo Fisher Scientific, Waltham, MA, USA) according to the manufacturer’s instructions. The protein samples (150–200 μg) were mixed with 2× reaction buffer containing 10 mM DTT and incubated at 37 °C for 2 h, followed by the addition of the DEVD-pNA substrate (200 μM). After the reaction, caspase-3 activity was measured by reading the absorbance at 400 nm using a microplate reader.

### Colony formation assay

To perform the colony formation assay, we followed previously reported methods [25] with slight modifications. SKOV-3 cells were plated (500 cells) in a 60-mm culture dish and cultured for 2 weeks. Thereafter, the cells were treated with the ER extract for 72 h and then fixed with 4% PFA. After fixation, the cells were stained with a solution containing 0.5% crystal violet (Sigma–Aldrich) in 25% methanol for 2 h, then washed, and observed.

### Subcellular fractionation and immunoblotting

Nuclear and cytosolic proteins were separately extracted from cells using a nuclear/cytosol fractionation kit (BioVision) according to the manufacturer’s manual. For immunoblotting, cells were treated with synephrine or limonin, with and without PFT-α, and then lysed in a lysis buffer containing 50 mM Tris–HCl (pH 7.5), 0.5% Triton X-100, 150 mM NaCl, 0.5 mM EGTA, and a protease inhibitor mixture (Roche, Basel, Switzerland). Protein concentrations in the cell lysates were determined using the BCA assay, and the proteins (20–30 μg) were mixed with an SDS sample buffer. The samples were boiled at 90 °C for 10 min and then separated by electrophoresis in 10–15% polyacrylamide gels. The separated proteins were transferred onto polyvinylidene difluoride membranes, which were blocked by incubation in TBST buffer with 5% nonfat milk or 5% bovine serum albumin (BSA) for 1 h. Afterward, the membranes were incubated with the following primary antibodies: p53 (1:1000; Abcam, Cambridge, UK), p65 (1:1000; Abcam), Bcl-2 (1:1000; Abcam), α-tubulin (1:5000; Cell Signaling Technology, Danvers, MA, USA), histone H3 (1:1000; Cell Signaling Technology), Bax (1:1000; Cell Signaling Technology), survivin (1:1000; Cell Signaling Technology), cleaved caspase-3 (1:1000; Cell Signaling Technology), and cleaved PARP1 (1:1000; Cell Signaling Technology) at 4 °C overnight. Next, the membranes were washed with TBST buffer, then incubated with horseradish peroxidase-conjugated secondary antibodies (1:5000; Cell Signaling Technology) at room temperature for 1 h, and washed again. Immunoreactive bands were detected using an enhanced chemiluminescence solution (Millipore, Burlington, MA, USA) in the dark.

### Immunofluorescence and imaging

For immunofluorescence microscopy, SKOV-3 cells were plated on coverslips, then treated with DMSO and limonin for 24 h, and fixed with 4% paraformaldehyde. The fixed cells were permeabilized with 0.2% Triton X-100, and the coverslips were blocked with 5% BSA. Afterward, the cells were incubated with primary antibodies against p53 (1:50; Abcam) and p65 (1:100; Abcam) for 1 h and washed with PBS, followed by incubation with Alexa 488-conjugated secondary antibodies. Subsequently, the coverslips were mounted on slides using a mounting solution containing DAPI, and the stained samples were imaged using a fluorescence microscope. Z-stack and live-cell images were acquired using a laser scanning confocal microscope (Zeiss 710 Meta; Zeiss, Oberkochen, Germany) and an optical microscope (CKX53; Olympus, Tokyo, Japan), respectively.

### Statistical analysis

The colony formation assay, colorimetric caspase-3 activity assay, cell viability assay, and immunoblotting data were obtained from at least three independent experiments. The colony formation assay and immunoblotting data were analyzed using ImageJ (http://rsb.info.nih.gov/ij), and all analyzed data were graphed using Origin Pro 8.0. For statistical analysis of differences between two groups, Student’s *t*-test was applied. The data are presented as the mean ± standard error of the mean (SEM). *P* < 0.05 was considered statistically significant.

## Results

### ER extract exerted anticancer effects in SKOV-3 cells

**Fig. 1:**
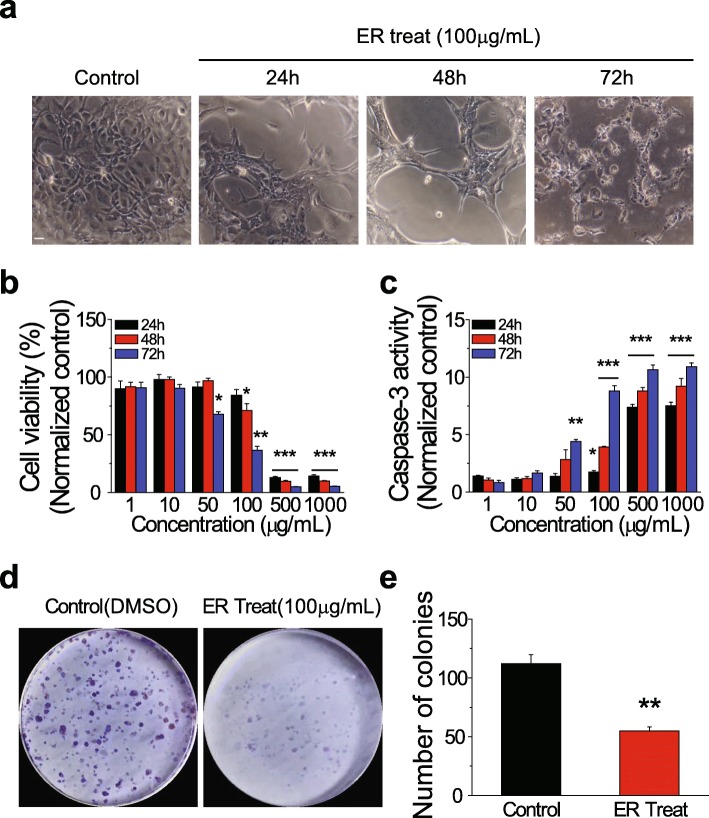
Effects of the *Evodia rutaecarpa* (ER) extract on viability of and caspase-3 activity in SKOV-3 ovarian cancer cells. (**a**) Representative images of cells treated with the control (DMSO) and the ER extract (100 μg/mL) for 24, 48, and 72 h. Scale bar = 10 μm. (**b**) Viability of and (**c**) caspase-3 activity in cells treated with the ER extract (1, 10, 50, 100, 500, and 1000 μg/mL) for 24, 48, and 72 h (mean ± SEM). (**d**) Representative images of colonies formed by control (DMSO)- and ER (100 μg/mL)-treated cells after 72 h of incubation. (**e**) Numbers of colonies (mean ± SEM). *P < 0.05, **P < 0.01, ***P < 0.001

We found that ER treatment reduced cell density and increased membrane blebbing in SKOV-3 cells (Fig. 1a). Membrane blebbing has been shown to indicate apoptosis [26]. To investigate the effect of the ER extract in ovarian cancer cells, the viability of ER-treated SKOV-3 cells was assessed and found to be significantly reduced in a concentration- and time-dependent manner (Fig. 1b). To examine whether the reduced viability of ER-treated cells was due to apoptosis, we measured caspase-3 activity. Compared with that in the control, ER-treated cells showed a significant increase in caspase-3 activation, in a concentration- and time-dependent manner (Fig. 1c). To further examine whether cell proliferation was inhibited by ER treatment, we performed a colony formation assay under the treatment conditions used for SKOV-3 cells, as discriminative cell viability and caspase-3 activity were observed under particular treatment conditions (for example, 100 μg of ER extract for 72 h). As expected, the number of colonies was significantly reduced by ER treatment compared with that in the control (Fig. 1d and e). Collectively, these results suggested that the ER extract exerted an anticancer effect via activation of caspase-3-dependent apoptosis in ovarian cancer cells.

### Limonin but not synephrine reduced the viability of SKOV-3, A2780, and RMUG-S cells

**Fig. 2:**
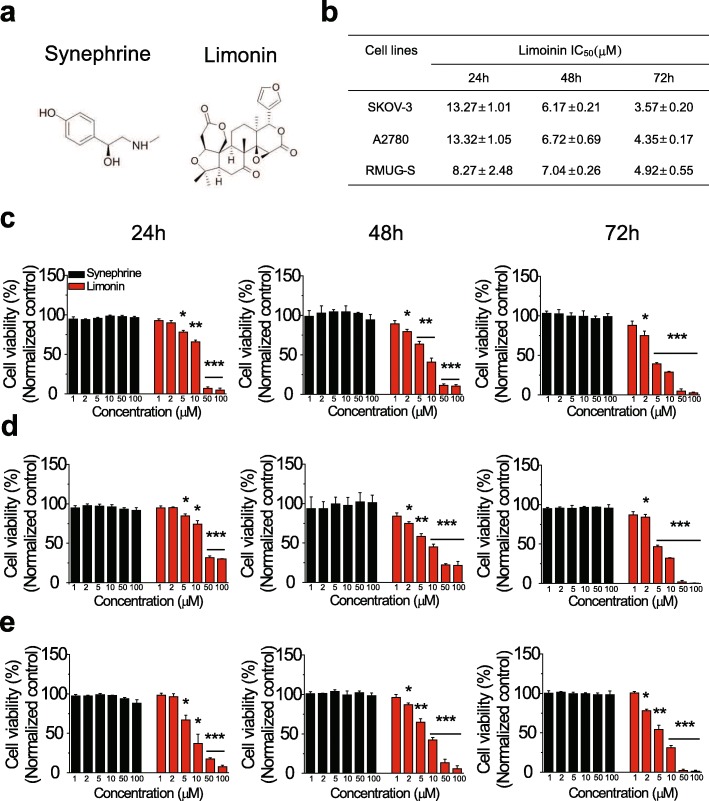
Effects of limonin and synephrine on various ovarian cancer cells. (**a**) Chemical structures of synephrine (left) and limonin (right). (**b**) IC_50_ values of limonin against ovarian cancer cell lines, A2780, SKOV-3, and RMUG-S. (**c–e**) Viability of human ovarian cancer cell lines, A2780 (**c**), SKOV-3 (**d**), and RMUG-S (**e**), treated with synephrine and limonin (1, 2, 5, 10, 50, and 100 μM) for 24, 48, and 72 h (mean ± SEM). *P < 0.05, **P < 0.01, ***P < 0.001

To determine the compound that contributed to the inhibitory effect of the ER extract in the ovarian cancer cell line, SKOV-3, we selected two ER-derived compounds, namely, synephrine and limonin (Fig. 2a), and assessed their effects on the viability of the SKOV-3, A2780, and RMUG-S ovarian cancer cell lines. Limonin-exposed cells showed a significant reduction in their viability in a concentration- and time-dependent manner; however, synephrine exhibited no effect (Fig. 2c–e). In addition, the IC_50_ values of limonin indicated that the mucinous ovarian cancer cell line, RMUG-S, was more susceptible to limonin than were the ovarian cancer cell lines of the serous-type at an early time point (Fig. 2b). Taken together, these results indicated that limonin inhibited the viability of the mucinous-type as well as the serous-type ovarian cancer cell lines, implying that the anticancer effect of the ER extract was due to limonin.

### Limonin changed the subcellular localization and level of the p53 protein

**Fig. 3:**
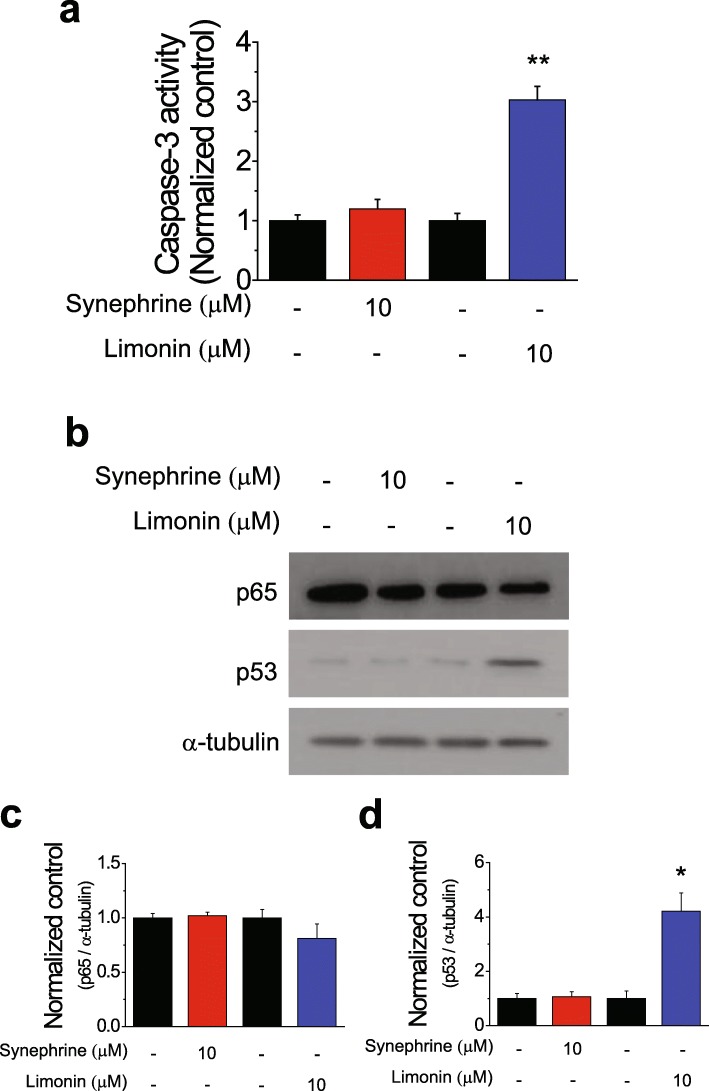
Molecular pathways involved in the induction of apoptosis by limonin. (**a**) Caspase-3 activity (mean ± SEM) in SKOV-3 cells treated with synephrine and limonin for 24 h. (**b**) Immunoblotting analysis of expression levels of p53 and p65 proteins in SKOV-3 cells treated with synephrine (10 μM), limonin (10 μM), or DMSO (control) for 24 h. (**c, d**) Quantification of p65 (**c**) and p53 (**d**) levels (mean ± SEM) in (**b**). α-Tubulin was used as a loading control. *P < 0.05

We further investigated whether limonin-induced cell death was due to caspase-3-dependent apoptosis of SKOV-3 cells. Based on the results of the cell viability assay, a concentration of 10 μM and an exposure time of 24 h were selected for the treatment with limonin. Limonin-treated cells exhibited a significantly enhanced caspase-3 activation compared with that in the control; as expected, synephrine-treated SKOV-3 cells did not exhibit any changes in the caspase-3 activity (Fig. 3a). p53 and p65 have been reported to be key molecules regulating the induction of apoptosis in human cancers [27, 28]. Thus, we examined whether the levels of p65 and p53 were affected by the treatment with limonin. The results of immunoblotting showed that the p53 level was significantly higher in limonin-treated SKOV-3 cells than in the control, whereas that of p65 was not different from its level in the control. Neither p53 nor p65 levels were affected by synephrine treatment (Fig. 3b–d).

**Fig. 4:**
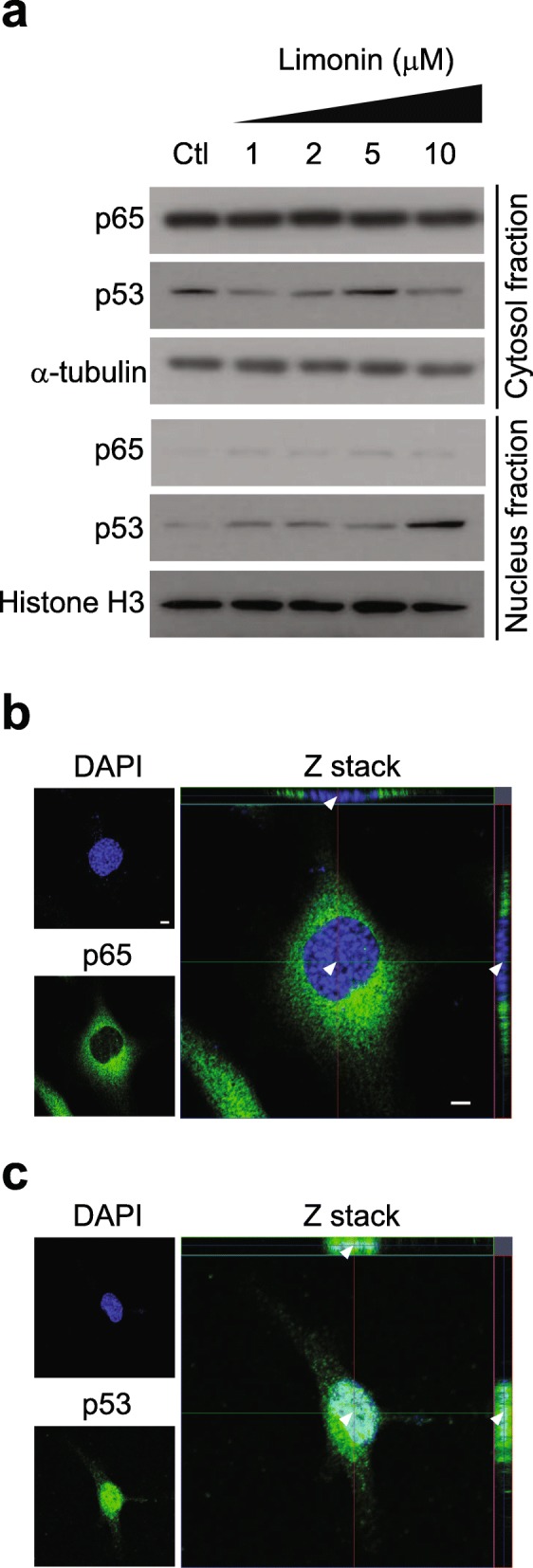
Effects of limonin on subcellular localization of the p53 protein. (**a**) Immunoblotting analysis of p65 and p53 protein levels in subcellular fractions of cells treated with increasing concentrations of limonin or with DMSO (control). α-Tubulin and histone H3 were used as loading controls for the cytosol and nuclear fractions, respectively. (**b, c**) Z-stack images of subcellular localization of p65 (**b**) and p53 (**c**) in SKOV-3 cells. Scale bar = 5 μm. The arrowheads indicate nuclear localization

Increased protein levels and nuclear p53 translocation have been implicated in the activation of p53 [29]. Therefore, we further investigated whether subcellular localization of p53 was affected by the treatment of SKOV-3 cells with different concentrations of limonin for 24 h. Subcellular localization of the p65 protein showed no change compared with that in the control; however, the p53 protein showed enhanced translocation from the cytosol to the nuclear fraction in SKOV-3 cells following limonin treatment (Fig. 4a). To confirm the localization of p53 in the nucleus, fixed cells were stained with labeled p65 and p53 antibodies following limonin treatment. A confocal Z-stack analysis revealed that signals from the stained p53 protein were merged with those of DAPI, indicating that the limonin treatment induced the translocation of the p53 protein to the nucleus, whereas the p65 protein was only localized to the cytosolic fraction of SKOV-3 cells (Fig. 4b and c). Taken together, these results indicated that limonin activated the p53 protein, increasing its level of expression and nuclear translocation.

### Limonin predominantly induced apoptosis of ovarian cancer cells via activation of p53

**Fig. 5:**
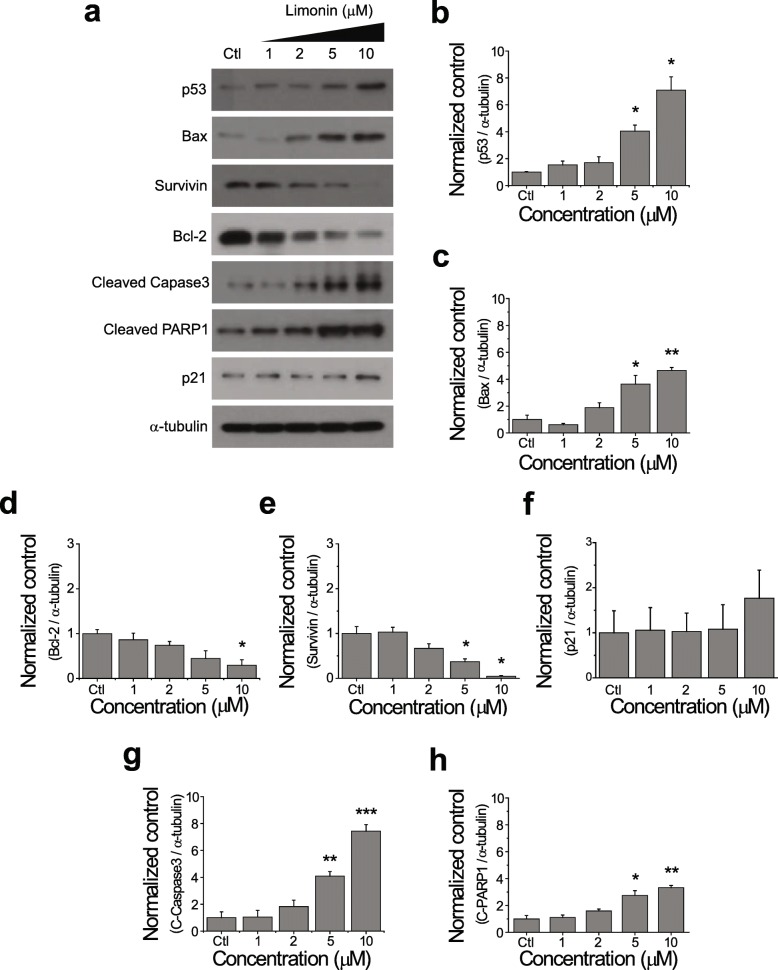
Activation of p53-mediated apoptosis by limonin in SKOV-3 cells. (**a**) Immunoblotting analysis of expression levels of proteins involved in the apoptotic pathway in cells treated with limonin (1, 2, 5, and 10 μM) or DMSO (control) for 24 h. (**b–h**) Quantification of p53 (**b**), Bax (**c**), Bcl-2 (**d**), survivin (**e**), p21 **(f**), cleaved caspase-3 (**g**), and cleaved PARP1 (**h**) levels (mean ± SEM) in (**a**). α-Tubulin was used as a loading control. *P < 0.05, **P < 0.01, ***P < 0.001

We performed immunoblotting to detect proteins involved in p53-mediated apoptosis pathway. Consistent with the previous results, limonin concentration-dependently increased the p53 protein level (Fig. 5a and b). Proapoptotic proteins, Bax, cleaved caspase-3, and PARP1, progressively increased in SKOV-3 cells treated with increasing concentrations of limonin (Fig. 5a, c, g, and h). On the contrary, the levels of antiapoptotic proteins, Bcl-2 and survivin, concentration-dependently decreased in limonin-treated cells (Fig. 5a, d, and e).

Previous studies have reported that functionally active p53 is implicated not only in the induction of apoptosis but also in cell-cycle arrest [28, 30, 31]. Thus, we next examined whether activation of p53 by limonin would also lead to cell-cycle arrest in ovarian cancer cells. The results showed that limonin treatment non-significantly elevated the level of the p21 protein (Fig. 5a and f), suggesting that activation of p53 by limonin predominantly affected the apoptotic pathway in SKOV-3 cells.

**Fig. 6:**
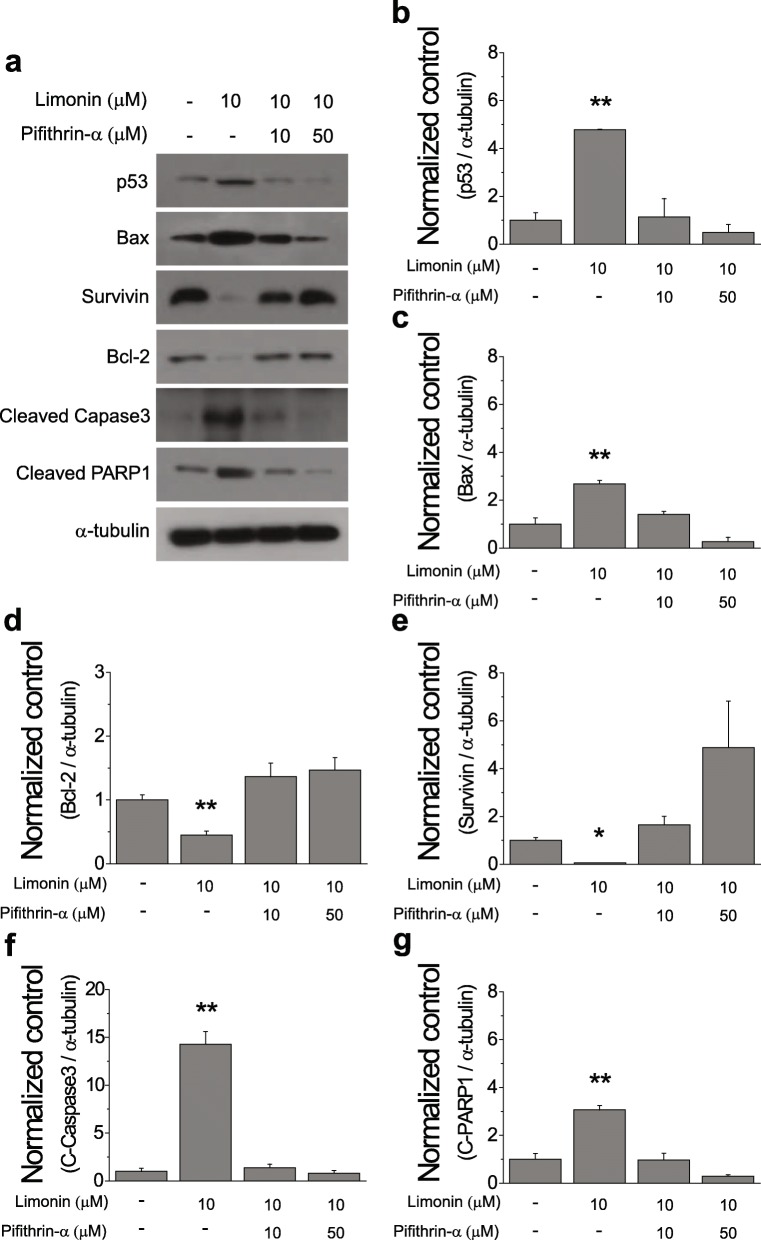
Effects of the p53 inhibitor PFT-α on limonin-induced apoptosis in the SKOV-3 ovarian cancer cell line. (**a**) Immunoblotting analysis of expression levels of proteins involved in the apoptotic pathway in cells treated with limonin alone (10 μM), limonin plus PFT-α (10 and 50 μM), and DMSO (control) for 24 h. (**b–g**) Quantification of p53 (**b**), Bax (**c**), Bcl-2 (**d**), survivin (**e**), cleaved caspase-3 (**f**), and cleaved PARP1 (**g**) levels (mean ± SEM) in (**a**). α-Tubulin was used as a loading control. *P < 0.05, **P < 0.01

To examine whether the activation of the p53-mediated apoptosis pathway by limonin would be inhibited by a p53 inhibitor, we treated SKOV-3 cells with limonin in the presence or absence of the p53 inhibitor PFT-α. The results showed that the increased levels of the proapoptotic proteins and the decreased levels of the antiapoptotic proteins were brought to their basal levels by the inhibition of the p53 activity (Fig. 6a–g). These findings demonstrated that activation of the p53 protein by limonin predominantly led to apoptotic cell death via the caspase-dependent pathway, rather than via cell-cycle arrest in ovarian cancer cells.

### Reversal of cisplatin resistance in ovarian cancer cells by limonin

**Fig. 7:**
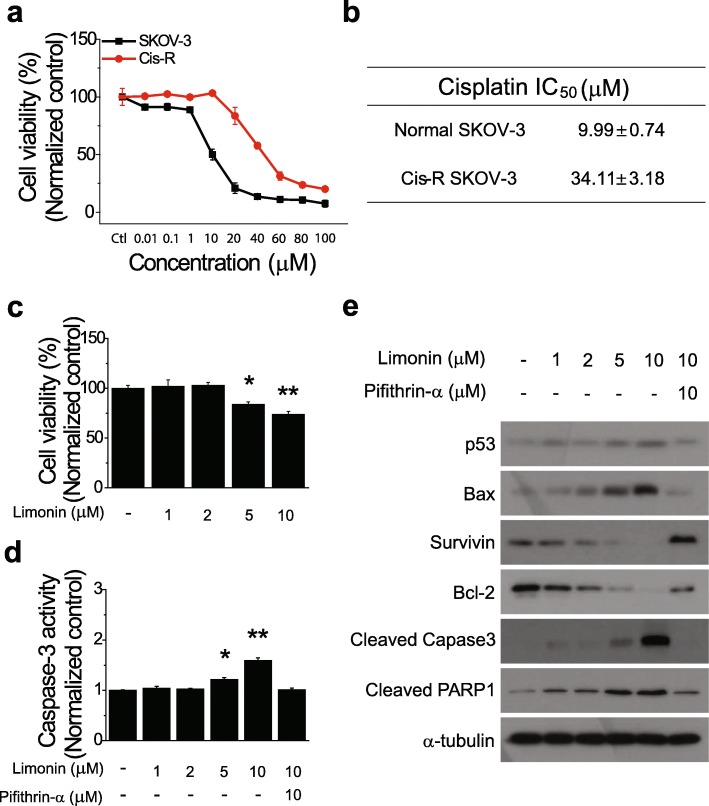
Effects of limonin on cisplatin resistance of ovarian cancer cells. **(a**) Viability of normal and cisplatin-resistant (Cis^R^) SKOV-3 cells treated with cisplatin (0.01–100 mM) for 72 h. (**b**) IC_50_ values of cisplatin against Cis^R^ and age-matched parental SKOV-3 cells. (**c**) Viability of Cis^R^ cells treated with limonin (1, 2, 5, and 10 μM). (**d**) Caspase-3 activity and (**e**) immunoblotting analysis of expression levels of proteins involved in the apoptotic pathway in Cis^R^ SKOV-3 cells exposed to limonin (1, 2, 5, and 10 μM) alone, limonin plus PTF-α (10 μM), or DMSO (control) for 24 h. α-Tubulin was used as a loading control. Data are expressed as the mean ± SEM. *P < 0.05, **P < 0.01

Chemoresistance has often been observed in ovarian cancer patients undergoing chemotherapy with platinum-based drugs [32]. Research on the development of new molecular targeted drugs has played an important role in overcoming resistance to platinum-based drugs. Indeed, a study has shown that the natural compound cryptotanshinone reversed resistance to cisplatin in lung cancer cells [33]. We next examined whether limonin could reverse cisplatin resistance of ovarian cancer cells. To this end, we generated a Cis^R^ ovarian cancer cell line via continuous exposure of normal SKOV-3 cells to cisplatin and evaluated the cisplatin sensitivity of the normal and Cis^R^ cell lines. The IC_50_ value of cisplatin for Cis^R^ SKOV-3 cells was 3.4-fold higher than that for normal SKOV-3 cells, confirming the cisplatin-resistant phenotype of the Cis^R^ SKOV-3 cell line (Fig. 7a and b).

Subsequently, we performed a cell viability assay to explore whether limonin could reverse the resistance of ovarian cancer cells to cisplatin. The results showed a significant, concentration-dependent reduction in the viability of limonin-treated Cis^R^ cells (Fig. 7c). To determine whether the reduction of Cis^R^ cell viability by the treatment with limonin was also due to apoptosis via activation of the p53-mediated pathway, Cis^R^ cells were treated with different concentrations of limonin, with and without PFT-α, for 24 h, and then the caspase-3 activity assay and immunoblotting were performed. Caspase-3 activity increased in limonin-treated Cis^R^ cells in a concentration-dependent manner; however, the increase was reversed by inhibition of p53 activity (Fig. 7d).

Consistent with the previous results, immunoblotting indicated that limonin concentration-dependently induced the activation of p53, increased the levels of proapoptotic proteins, and decreased those of antiapoptotic proteins. Furthermore, the activation of p53-mediated apoptosis by limonin was inhibited by the treatment of Cis^R^ cells with the p53 inhibitor (Fig. 7e). Collectively, these results indicated that limonin reversed the resistance of Cis^R^ ovarian cancer cells to cisplatin via the activation of the p53-mediated apoptosis pathway.

## Discussion

Natural products and their derivatives are gaining interest as novel therapeutic agents to treat several types of cancer because these substances can exert multiple anticancer effects and are associated with few side effects, unlike existing anticancer drugs. Among medicinal plants, ER has been reported in various studies to show anticancer effects [11, 12]. Several studies on ER derivatives have already shown that evodiamine has an anticancer effect in ovarian cancer cells [34, 35]. We selected limonin and synephrine as novel therapeutic candidates, potentially contributing to the effect of ER, because these compounds have not yet been investigated against ovarian cancer.

Our results indicated that limonin but not synephrine specifically inhibited the growth of ovarian cancer cells (Fig. 2). Further, limonin exerted its effects by enhancing caspase-3 activity in ovarian cancer cells, indicating the induction of apoptosis. The process of apoptosis is tightly regulated through pathways related to two major molecules, p53 and p65 proteins, in human cancers. We investigated the effect of limonin on the cellular levels of p53 and p65 proteins in ovarian cancer cells and found that the limonin-induced apoptotic cell death was initiated by the activation of the p53 protein, subsequently leading to an increase in the level of p53 as well as to its nuclear translocation, thereby resulting in the activation of proteins involved in the process of apoptosis (Figs. 3 and 4).

**Fig. 8:**
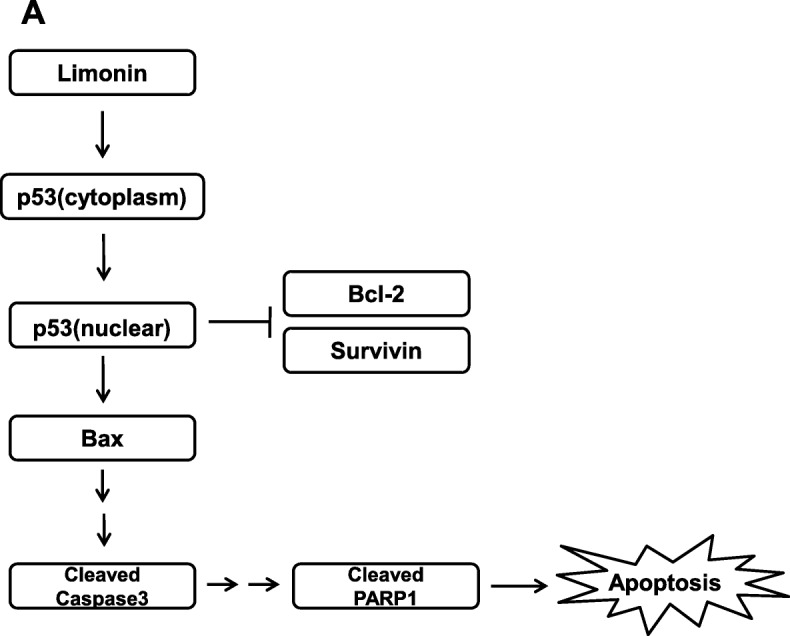
Mechanism of anticancer effects of limonin in ovarian cancer

Accumulating evidence suggests an anticancer effect of limonin via p53-mediated apoptosis. A study has shown that limonin upregulated the mRNA and protein levels of p53 in a hepatoma cell line, HepG2 [23]. Moreover, apoptotic cell death via upregulation of proapoptotic proteins and downregulation of antiapoptotic proteins by limonin treatment was commonly observed in pancreatic, breast, colon, and liver cancer cell lines [18–22]. These results are consistent with our findings, indicating that limonin exerted anticancer effects via activation of the p53-mediated apoptosis pathway in ovarian cancer cells (Fig. 8).

In our study, the mucinous-type ovarian cancer cell line RMUG-S was found to be more susceptible to limonin than were the serous types, SKOV-3 and A2780, at an early time point (Fig. 2). Several studies have provided evidence for clear differences in protein expression between serous and mucinous cell types in ovarian cancer [36–38]. For example, it has been shown that mucinous cell types are more likely to express E-cadherin than serous cell types are [37]. In addition, increased expression levels of MMPs, including MMP-2, MMP-7, and MMP-9, as well as TIMPs, including TIMP-1 and TIMP-2, were observed in mucinous ovarian cancer compared with those in serous ovarian cancer [38]. In particular, increased activity of MMP-2 and decreased expression of E-cadherin were associated with tumor growth and development [39, 40]. The activity of MMP-2 and the expression of E-cadherin have been reported to be modulated by p53 [41, 42]. In addition, a previous study has reported that the rates of p53 mutations in the serous and mucinous types of ovarian cancer were 56 and 17%, respectively [43], indicating a significantly higher p53 mutation rate in the serous-type than in the mucinous-type ovarian cancer. In this respect, we suggest that the activation of p53 by limonin in ovarian cancer cells may have affected the susceptibility of the mucinous ovarian cancer cell line via an unknown mechanism, related to the difference in protein expression between the ovarian cancer cell types. Further, the mucinous-type often exhibits resistance to platinum-based drugs during chemotherapy in the advanced stage [44]. Our results indicated that limonin reversed the resistance to cisplatin in ovarian cancer cells via activation of p53-mediated apoptosis (Fig. 7), suggesting that limonin may potentially be a novel strategic drug for chemotherapy of mucinous-type ovarian cancer.

We did not measure the content of limonin in the ER extract because we focused on the mechanism of the anticancer effect of ER and limonin. However, a previous study has reported that the content of limonin in ER was no less than 0.50% [14].

## Conclusions

In conclusion, we demonstrated that limonin contributed to the anticancer effect of ER via activation of p53-mediated apoptosis in ovarian cancer cells. Furthermore, we showed that limonin affected both serous and mucinous types of ovarian cancer and reversed the resistance of ovarian cancer cells to cisplatin. Limonin could be an attractive therapeutic agent for ovarian cancer.

## Data Availability

The data that support the findings of this study are available from the corresponding author upon reasonable request.

## Abbreviations

BSA: Bovine serum albumin
Cis^R^: Cisplatin-resistant
ER: *Evodia rutaecarpa*
PFT-α: Pifithrin-α
SEM: Standard error of the mean
